# Proton Pump Inhibitors and Hospitalization with Hypomagnesemia: A Population-Based Case-Control Study

**DOI:** 10.1371/journal.pmed.1001736

**Published:** 2014-09-30

**Authors:** Jonathan Zipursky, Erin M. Macdonald, Simon Hollands, Tara Gomes, Muhammad M. Mamdani, J. Michael Paterson, Nina Lathia, David N. Juurlink

**Affiliations:** 1 Department of Medicine, Sunnybrook Health Sciences Centre, Toronto, Ontario, Canada; 2 Institute for Clinical Evaluative Sciences, Toronto, Ontario, Canada; 3 Keenan Research Centre of the Li Ka Shing Knowledge Institute, St. Michael's Hospital, Toronto, Ontario, Canada; 4 Institute of Health Policy, Management, and Evaluation, University of Toronto, Toronto, Ontario, Canada; 5 Leslie Dan Faculty of Pharmacy, University of Toronto, Toronto, Ontario, Canada; 6 Applied Health Research Centre, St. Michael's Hospital, Toronto, Ontario, Canada; 7 King Saud University, Riyadh, Saudi Arabia; 8 Department of Family Medicine, McMaster University, Hamilton, Ontario, Canada, Canada; 9 Sunnybrook Research Institute, Toronto, Ontario, Canada; University of Pennsylvania School of Medicine, United States of America

## Abstract

**Background:**

Some evidence suggests that proton pump inhibitors (PPIs) are an under-appreciated risk factor for hypomagnesemia. Whether hospitalization with hypomagnesemia is associated with use of PPIs is unknown.

**Methods and Findings:**

We conducted a population-based case-control study of multiple health care databases in Ontario, Canada, from April 2002 to March 2012. Patients who were enrolled as cases were Ontarians aged 66 years or older hospitalized with hypomagnesemia. For each individual enrolled as a case, we identified up to four individuals as controls matched on age, sex, kidney disease, and use of various diuretic classes. Exposure to PPIs was categorized according to the most proximate prescription prior to the index date as current (within 90 days), recent (within 91 to 180 days), or remote (within 181 to 365 days). We used conditional logistic regression to estimate the odds ratio for the association of outpatient PPI use and hospitalization with hypomagnesemia. To test the specificity of our findings we examined use of histamine H2 receptor antagonists, drugs with no causal link to hypomagnesemia. We studied 366 patients hospitalized with hypomagnesemia and 1,464 matched controls. Current PPI use was associated with a 43% increased risk of hypomagnesemia (adjusted odds ratio, 1.43; 95% CI 1.06–1.93). In a stratified analysis, the risk was particularly increased among patients receiving diuretics, (adjusted odds ratio, 1.73; 95% CI 1.11–2.70) and not significant among patients not receiving diuretics (adjusted odds ratio, 1.25; 95% CI 0.81–1.91). We estimate that one excess hospitalization with hypomagnesemia will occur among 76,591 outpatients treated with a PPI for 90 days. Hospitalization with hypomagnesemia was not associated with the use of histamine H2 receptor antagonists (adjusted odds ratio 1.06; 95% CI 0.54–2.06). Limitations of this study include a lack of access to serum magnesium levels, uncertainty regarding diagnostic coding of hypomagnesemia, and generalizability of our findings to younger patients.

**Conclusions:**

PPIs are associated with a small increased risk of hospitalization with hypomagnesemia among patients also receiving diuretics. Physicians should be aware of this association, particularly for patients with hypomagnesemia.

*Please see later in the article for the Editors' Summary*

## Abstract 2

David Juurlink and colleagues evaluated the risk of hospitalization with hypomagnesemia among patients taking proton pump inhibitors.

*Please see later in the article for the Editors' Summary*

## Editors' Summary

**Background:**

To extract nutrients from food, we rely on a multi-stage process called digestion. A crucial stage in digestion occurs in the stomach where gastric juice, a mixture of mainly hydrochloric acid and the enzyme pepsin, breaks down the proteins present in food. We could not digest food without gastric juice, but the acid it contains, which is made by glands in the stomach, can damage the lining of the digestive system and cause symptoms of indigestion (dyspepsia), stomach (peptic) ulcers, and gastroesophageal reflux disease (GERD), a condition in which acid from the stomach leaks back up the esophagus (gullet), Acid-related disorders are often treated with proton pump inhibitors (PPIs), a class of drugs that reduces acid production in the stomach. Omeprazole, lansoprazole, and other PPIs are among the most widely prescribed drugs in the world. In 2010, 147 million prescriptions for PPIs were dispensed in the US alone.

**Why Was This Study Done?:**

Like all drugs, PPIs have some unwanted side effects. They sometimes cause diarrhea, for example, and their long-term use is associated with fractures. In addition, long-term PPI use may be a risk factor for hypomagnesemia, a condition in which the magnesium level in the blood is abnormally low. If severe, hypomagnesemia can lead to life-threatening heart arrhythmias and seizures (fits). Magnesium levels are controlled by absorption of magnesium by the intestines and excretion of magnesium by the kidneys. It is thought that PPI-related hypomagnesemia involves inhibition of magnesium absorption. Given the widespread use of PPIs, it is important to know whether PPIs are a risk factor for hypomagnesemia in routine clinical practice. In this population-based case-control study, the researchers ask whether hospitalization with hypomagnesemia is associated with the use of PPIs. A case-control study compares the characteristics of individuals with a specific condition with those of matched controls without the condition.

**What Did the Researchers Do and Find?:**

The researchers identified everyone aged 66 years or older who received a diagnosis of hypomagnesemia following hospital admission in Ontario over a 10 year period (366 cases) by searching a large database of hospital admissions. They identified up to four control patients from the general population who were matched with these case patients on age, sex, kidney disease, and the use of various diuretic classes (diuretic use is also associated with hypomagnesemia), and obtained data on PPI use by all patients from a database that records the prescription drugs dispensed to elderly Ontario residents. The researchers then used statistical methods to look for associations between current PPI use (a prescription within 90 days of the index date) and hospitalization with hypomagnesemia. After allowing for other characteristics that increase the risk of hypomagnesemia (including other illnesses), current PPI use was associated with a 43% increased risk of hypomagnesemia. Among patients receiving diuretics, PPI use increased the risk of hypomagnesemia by 73% whereas among patients not receiving diuretics, PPI use did not significantly increase the risk of hypomagnesemia. Finally, the researchers calculated that 76,591 individuals would need to be treated with a PPI as an outpatient for 90 days to result in one additional hospitalization with hypomagnesemia.

**What Do These Findings Mean?:**

These findings show that, among elderly individuals, current (but not previous) outpatient use of PPIs is associated with an increased risk of detection of hypomagnesemia during hospitalization, particularly among patients also taking diuretics. Because this study only considered elderly patients, these findings may not apply to younger patients. Moreover, the accuracy of these findings may be affected by the validity of the hospital coding for hypomagnesemia in the database used to identify cases. Importantly, given the large number of patients that need to take PPIs to result in one additional hospitalization with hypomagnesemia, these findings should not discourage clinicians from prescribing PPIs to appropriate patients nor should they lead to calls for routine screening of magnesium levels in patients taking PPIs. Rather, these findings highlight the need for clinicians to be aware of the association between PPI use and the risk of hypomagnesemia and to reassess ongoing therapy in patients who develop hypomagnesemia while taking PPIs.

**Additional Information:**

Please access these websites via the online version of this summary at http://dx.doi.org/10.1371/journal.pmed.1001736.

- The UK National Health Service Choices website provides information about symptoms, causes, and treatment of indigestion, heartburn and gastroesophageal reflux disease, and stomach ulcers; a “Behind the Headlines” article from 2010 discusses an editorial about the possible over-use of PPIs
- MedlinePlus provides links to information about indigestion, stomach ulcer, and gastroesophageal reflux disease (in English and Spanish); the MedlinePlus encyclopedia has pages on proton pump inhibitors (in English and Spanish) and on hypomagnesemia (in English and Spanish)
- A US Federal Drug Agency warning about the possible association between proton pump inhibitors and hypomagnesemia is available
- Wikipedia pages on proton pump inhibitors and on hypomagnesemia are also available (note that Wikipedia is a free online encyclopedia that anyone can edit; available in several languages)

## Introduction

Proton-pump inhibitors (PPIs) are among the most widely prescribed drugs in the world, with more than 147 million prescriptions dispensed in the United States in 2010 alone [1]. They are the mainstay of drug therapy for acid-related disorders, and have largely supplanted histamine H2 receptor antagonists owing to their superior efficacy [2]–[4].

Although widely regarded as safe, PPIs have been associated with a variety of adverse effects including *Clostridium difficile*-associated diarrhea [5]–[7], interstitial nephritis [8],[9], pneumonia [10], vitamin B12 deficiency [11], and osteoporosis and fractures [12]. More recently, long-term use of PPIs has been suggested as a potential cause of hypomagnesemia [13]. Magnesium is the second most abundant intracellular cation and its homeostasis is intricately regulated by intestinal absorption and renal excretion, although the estimated prevalence of hypomagnesemia in the general population ranges from 2.5% to 15% [14],[15]. The postulated mechanism of PPI-related hypomagnesemia involves inhibition of intestinal magnesium absorption via transient receptor potential melastin (TRPM) 6 and 7 cation channels [16]. Severe hypomagnesemia can be associated with malignant cardiac arrhythmias, tetany, generalized seizures, and other metabolic disturbances such as hypokalemia and hypocalcemia [17].

First described in 2006 [18], the evidence for PPI-induced hypomagnesemia has been mainly limited to case reports and small case series, with more than 30 cases published to date [18]–[30]. In 2011, the US Food and Drug Administration (FDA) issued a drug safety warning regarding the potential association of PPIs with hypomagnesemia, but observational studies have yielded conflicting findings [31]–[34]. The largest study to date evaluated patients in a single intensive care unit, and found that PPI use was associated with hypomagnesemia only among patients also receiving diuretics [31]. It is uncertain whether PPIs are a risk factor for hypomagnesemia in routine clinical practice.

Given the widespread use of PPIs, particularly in combination with other drugs that can promote hypomagnesemia, such an association may be difficult to appreciate clinically. We examined the association between outpatient PPI use and hospitalization with hypomagnesemia.

## Methods

### Ethics Statement

This study was approved by the Research Ethics Board of Sunnybrook Health Sciences Centre. The Institute for Clinical Evaluative Sciences (ICES) is named as a prescribed entity under section 45 of the *Personal Health Information Protection Act* (Ontario Regulation 329/04, Section 18). Under this designation, ICES can receive and use anonymous health information without consent.

### Setting

We conducted a population-based case-control study of all Ontario residents aged 66 years or older between April 1st, 2002 and March 31st, 2012. These individuals had universal access to physician services, hospital care, and prescription drug coverage.

### Data Sources

We identified prescription records using the Ontario Drug Benefit Database, which contains comprehensive records of prescription drugs dispensed to Ontario residents aged 65 years or older. To avoid incomplete medication records, we excluded patients during their first year of eligibility for prescription drug coverage (age 65). We obtained hospitalization data from the Canadian Institute for Health Information Discharge Abstract Database, which contains detailed clinical information, including diagnoses, for all hospital admissions in Ontario. Emergency department records were obtained from the National Ambulatory Care Reporting System. We used the Ontario Health Insurance Plan database to identify claims for physician services, the Ontario Diabetes Database [35] to ascertain the presence of diabetes, and the Ontario Congestive Heart Failure Database [36] to identify individuals with congestive heart failure. We obtained basic demographic data and date of death from the Registered Persons Database, a registry of all Ontario residents eligible for health insurance. These databases were linked in an anonymous fashion using encrypted health card numbers, and are routinely used to study drug safety [37]–[39].

### Study Patients

We defined case patients as those hospitalized with hypomagnesemia, defined using the International Classification of Diseases and Related Health Problems, Tenth Revision (ICD-10) codes E83.42 (hypomagnesemia) or E61.2 (magnesium deficiency). Only the first such hospitalization was considered for patients with multiple episodes. The date of hospital admission served as the index date for all analyses. For each individual enrolled as a case, we randomly selected four control patients not hospitalized with hypomagnesemia. Control patients were randomly assigned an index date within one calendar year of the corresponding case patient, and patients who were controls could later serve as cases. Four control patients were matched to each case patient according to age (within 3 years), sex, chronic kidney disease (CKD), or acute kidney injury (AKI) in the year preceding the index date, and receipt of thiazide, loop, or other diuretics in the 90 days preceding the index date, with each diuretic class considered separately. Each individual could only serve once as a control and unmatched cases were excluded. We also excluded patients with a diagnosis of hyperparathyroidism or inflammatory bowel disease in the year prior to index date because these disorders can influence magnesium balance, and we excluded individuals hospitalized for any reason in the month preceding the index date to avoid the potential confounding effects of recent hospitalization.

### Assessment of PPI Exposure

We identified all outpatient prescriptions for omeprazole, lansoprazole, pantoprazole, rabeprazole, and esomeprazole. We classified PPI exposure according to the prescription closest to the index date as either current (within 90 days preceding the index date), recent (91 to 180 days prior to the index date), or remote (181 to 365 days prior to the index date). We used this approach because many patients take PPIs intermittently, particularly for symptoms of dyspepsia or gastroesophageal reflux. Moreover, any association should manifest in patients with ongoing PPI therapy, and would therefore be most likely in current users and least likely in remote users [13]. As well, in many published reports, magnesium levels have normalized shortly after discontinuation of the PPI [13].

To test the robustness and specificity of our findings, we also examined prescriptions for histamine H2 receptor antagonists, drugs with no plausible causal link to hypomagnesemia.

### Statistical Analysis

We used conditional logistic regression to estimate the odds ratio and 95% confidence intervals for the association between hypomagnesemia and receipt of a PPI prescription. In all analyses, the reference group consisted of patients with no PPI prescription in the 365 days preceding the index date. A similar analysis was conducted for histamine H2 receptor antagonists. A subgroup analysis was performed to examine the risk of hypomagnesemia associated with PPI use amongst patients concomitantly prescribed diuretics.

We adjusted all models for confounders with standardized differences>0.10 including the number of drugs dispensed in the year preceding the index date (a validated measure of comorbidity) [40], systemic steroid use in the preceding year, any history of diabetes or heart failure in the 3 years prior to the index date, and the presence of systemic malignancy in the preceding year. We used SAS version 9.3 for all analyses (SAS Institute), and a two-tailed type 1 error rate of 0.05 as the threshold for statistical significance.

## Results

During the ten-year study period, we identified 429 patients aged 66 years or older hospitalized with hypomagnesemia. We excluded 63 patients enrolled as cases (14.7%) who had been hospitalized in the prior month or had a diagnosis of inflammatory bowel disease in the previous year. The remaining 366 case patients were matched to 1,464 patients designated as controls. The characteristics of case and control patients are shown in Table 1. The median age was 78 (interquartile range [IQR] 71–83) years, and slightly more than half were women. As expected, compared with control patients, case patients received more medications and were more likely to have various comorbid conditions (Table 1).

**Table 1:** Characteristics of patients enrolled as cases and controls.

Characteristic	Case Patients (*n* = 366)	Control Patients (*n* = 1,464)	Standardized Difference
Median (IQR) age (years)	78 (71–83)	78 (71–83)	0
Age group (years)			
66–75	149 (40.7)	596 (40.7)	0
76–85	159 (43.4)	636 (43.4)	0
86+	58 (15.5)	232 (15.8)	0
Male	145 (39.6)	580 (39.6)	0
Income quintile			
1 (lowest)	73 (19.9)	296 (20.2)	0.01
2	67 (18.3)	308 (21.0)	0.07
3	81 (22.1)	282 (19.3)	0.07
4	73 (19.9)	264 (18.0)	0.05
5	70 (19.1)	264 (18.0)	0.03
Missing	≤5^a^	50 (3.4)	0.17
Residence in LTC	32 (8.7)	103 (7.0)	0.07
Median (IQR) number of distinct drugs used in previous year^b^	12 (7–16)	7 (3–11)	0.69
Diabetes	26 (7.1)	65 (4.4)	0.12
Congestive heart failure	45 (12.3)	84 (5.7)	0.26
Liver disease	6 (1.6)	11 (0.8)	0.09
Systemic malignancy	49 (13.4)	47 (3.2)	0.46
Alcoholism	10 (2.7)	≤5^a^	0.32
Hypertension	22 (6.0)	82 (5.6)	0.02
Steroid use	54 (14.8)	116 (7.9)	0.24
CKD in one year prior	32 (8.7)	128 (8.7)	0
Loop diuretics^c^	71 (19.4)	284 (19.4)	0
Thiazide diuretics^c^	72 (19.7)	288 (19.7)	0
Other diuretics^c^	17 (4.6)	68 (4.6)	0

a Cell sizes lower than 6 are suppressed in accordance with institutional privacy regulations.

b Number of distinct drugs is a surrogate marker for comorbidity.

c Prescription in the prior 90 days.

CKD, chronic kidney disease; LTC, long term care.

In the primary analysis, we found that patients hospitalized with hypomagnesemia were more likely than control patients to be current users of PPIs (adjusted odds ratio, 1.43; 95% CI 1.06–1.93). Neither recent nor remote PPI use was associated with a significantly increased risk of hypomagnesemia (Table 2). The median number of tablets dispensed to patients who were cases in the 5 years preceding the index date was 1,358 (IQR 540–1,829) versus 1,176 (IQR 540–1,831) among patients who were controls. As expected, we found no association between hospitalization with hypomagnesemia and current use of histamine H2 receptor antagonists (unadjusted odds ratio 2.12, 95% CI 1.17–3.86; adjusted odds ratio 1.06; 95% CI 0.54–2.06) (Table 3). In a subgroup analysis of diuretic users, there was a higher risk of hospitalization with hypomagnesemia in patients using PPIs who were also concomitantly prescribed diuretics (adjusted odds ratio, 1.73; 95% CI 1.11–2.70) (Table 4).

**Table 2:** Proton pump inhibitor use and hospitalization with hypomagnesemia.

Characteristic	Number (%) of Patients with Exposure		Odds Ratio (95% Confidence Interval)	
	Case Patients (*n* = 366)	Control Patients (*n* = 1,464)	Unadjusted	Adjusted^a^
≤90 days (current)^b^	143 (39.1)	305 (20.8)	2.70 (2.08–3.51)	1.43 (1.06–1.93)
91–180 days (recent)^b^	12 (3.3)	42 (2.9)	1.59 (0.82–3.07)	1.03 (0.51–2.06)
181–365 (remote)^b^	9 (2.5)	35 (2.4)	1.37 (0.65–2.89)	0.94 (0.42–2.11)
Number of distinct drugs used in previous year	—	—	1.13 (1.11–1.16)	1.12 (1.09–1.14)
Diabetes	26 (7.1)	65 (4.4)	1.66 (1.03–2.67)	1.45 (0.85–2.47)
Congestive heart failure	45 (12.3)	84 (5.7)	2.64 (1.73–4.03)	1.70 (1.06–2.73)
Malignancy	49 (13.4)	47 (3.2)	4.71 (3.07–7.22)	3.52 (2.20–5.65)
Steroid use	54 (14.8)	116 (7.9)	2.01 (1.42–2.85)	0.97 (0.65–1.46)

a Adjusted for diabetes, congestive heart failure, malignancy, steroid use, and number of distinct drugs used in the past year (excluding PPI).

b Includes omeprazole, pantoprazole, lansoprazole, esomeprazole, and lansoprazole.

**Table 3:** Histamine (H2) receptor blocker use and hospitalization with hypomagnesemia.

Timing of Most Recent H2 Blocker Prescription^a^	Number (%) of Patients with Exposure		Odds Ratio (95% Confidence Interval)	
	Case Patients (*n* = 202)	Control Patients (*n* = 808)	Unadjusted	Adjusted^b^
≤90 days (current)	19 (9.4)	41 (5.1)	2.12 (1.17–3.86)	1.06 (0.54–2.06)
91–180 days (recent)	7 (3.5)	7 (0.9)	4.14 (1.45–11.83)	3.23 (0.98–10.62)
181–365 (remote)	6 (3.0)	13 (1.6)	2.11 (0.77–5.81)	1.27 (0.44–3.71)

a Includes cimetidine, ranitidine, nizatidine, and famotidine.

b Adjusted for diabetes, congestive heart failure, malignancy, steroid use, and number of distinct drugs used in the past year (excluding PPI).

**Table 4:** Proton pump inhibitor use and hospitalization with hypomagnesemia stratified by diuretic use.

Subgroup	Number (%) of Patients with Exposure		Odds Ratio (95% Confidence Interval)	
	Case Patients (*n* = 345)	Control Patients (*n* = 1,305)	Unadjusted	Adjusted^a^
**No diuretic**				
No PPI	139 (65.6)	672 (84.3)	1.0 (ref)	1.0 (ref)
Current PPI use	73 (34.4)	125 (15.7)	2.96 (2.08–4.22)	1.25 (0.81–1.91)
Number of distinct drugs used in previous year	—	—	1.15 (1.12–1.19)	1.13 (1.10–1.17)
Diabetes	14 (6.6)	26 (3.3)	2.10 (1.06–4.15)	1.59 (0.71–3.56)
Congestive heart failure	17 (8.0)	12 (1.5)	6.45 (2.85–14.61)	2.41 (0.94–6.20)
Malignancy	30 (14.2)	24 (3.0)	5.44 (3.01–9.84)	3.69 (1.88–7.24)
Steroid use	30 (14.2)	41 (5.1)	3.11 (1.86–5.22)	1.21 (0.66–2.22)
**Diuretic**				
No PPI	63 (47.4)	350 (68.9)	1.0 (ref)	1.0 (ref)
Current PPI use	70 (52.6)	158 (31.1)	2.52 (1.68–3.77)	1.73 (1.11–2.70)
Number of distinct drugs used in previous year	—	—	1.11 (1.07–1.15)	1.09 (1.05–1.14)
Diabetes	12 (9.0)	32 (6.3)	1.51 (0.75–3.04)	1.53 (0.72–3.29)
Congestive heart failure	24 (18.0)	61 (12.0)	1.69 (0.98–2.93)	1.34 (0.74–2.44)
Malignancy	17 (12.8)	17 (3.4)	3.92 (1.98–7.79)	3.48 (1.66–7.27)
Steroid use	20 (15.0)	63 (12.4)	1.24 (0.72–2.12)	0.74 (0.40–1.36)

a Adjusted for diabetes, congestive heart failure, malignancy, steroid use, and number of distinct drugs used in the past year (excluding PPI).

We anticipated that hospitalization with hypomagnesemia would be uncommon among patients treated with a PPI. Among 1,042,765 patients who commenced treatment with a PPI during the study period, 15 were hospitalized with hypomagnesemia in the subsequent 90 days, representing 3.19 such admissions (95% CI 1.95–5.2) per 100,000 new PPI prescriptions. Among all eligible control patients (*n* = 1,040,875), fewer than six individuals were admitted with hypomagnesemia within 90 days, yielding a number needed to harm at 90 days of 76,591.

## Discussion

We found that current PPI therapy was associated with a 43% increased relative risk of hospitalization with hypomagnesemia in a large population of older outpatients. In contrast, no such risk was evident with more remote use of PPIs or of histamine H2 receptor antagonists. Although mild hypomagnesemia may go unnoticed, severe cases can have significant neurologic and cardiac consequences. Our findings highlight an underappreciated albeit small risk of these commonly prescribed medications.

In an analysis stratified by diuretic use, concomitant users of PPIs and diuretics had an increased risk of hospitalization with hypomagnesemia, whereas those not taking diuretics did not have an increased risk. This finding accords with that of Danziger and colleagues, who studied the association between PPIs and hypomagnesemia in an intensive care unit setting [31]. While PPI-induced hypomagnesemia is rare and most PPI users are presumably unaffected, patients taking diuretics may be at particular risk.

Previous studies examining the association between PPIs and hypomagnesemia have found conflicting results. These studies [32]–[34] did not completely adjust for comorbidity and polypharmacy, important confounding factors contributing to hypomagnesemia, and two studies were limited to hospital inpatients [31],[32]. Another recent case-control study by Koulouridis and colleagues found no association between outpatient PPI use and hypomagnesemia at hospital admission [33]. Some reasons why this study's conclusion may have differed from ours include the potential for misclassification of PPI use, because physician and nursing medication administration records were the primary methods of ascertaining exposure, with specificity documented to be as low as 46.2%. In contrast, we examined population-based prescriptions records and accounted for timing of the prescription. Their definition (serum magnesium concentration <1.4 mEq/l) almost certainly included less severe cases of hypomagnesemia than in our study, which examined hospitalization with hypomagnesemia—a less sensitive but more clinically meaningful outcome. Finally, their study was limited to patients at a single center hospitalized for disorders of the upper gastrointestinal tract. The largest study to date evaluated hypomagnesemia in a single intensive care unit and found that PPIs were only associated with hypomagnesemia (serum magnesium concentration <1.6 mg/dl) in patients with concurrent use of diuretics [31]. While this study supports the notion that PPIs may be a risk factor for hypomagnesemia, the generalizability of these findings to patients in routine clinical practice is uncertain.

The mechanism of hypomagnesemia in the setting of PPI use is poorly understood but may reflect impaired gastrointestinal absorption of magnesium. TRPM 6 and 7 cation channels in the intestine are responsible for active transport of magnesium [16]. In contrast, most other drugs associated with hypomagnesemia cause renal magnesium wasting. In patients with suspected PPI-induced hypomagnesemia, renal magnesium handling is preserved [20],, although as found in our study and by others [31], this may be influenced by diuretic therapy.

We speculate that hypomagnesemia is an underappreciated consequence of PPI use, particularly given the high background use of PPIs in the general population. Whether some PPI recipients are genetically predisposed to this adverse effect is currently unknown. It has been postulated that individuals carrying heterozygous mutations of TRPM6/7 may be at especially high risk, as evidenced from observations that people with homozygous mutations of TRPM6 manifest severe hypomagnesemia [42]–[44]. The modest effect found in this study may in fact reflect a much larger problem with systemic magnesium balance, since only about 1% of total body magnesium is reflected in the blood [45].

The findings of this study are strengthened by the population-based nature of the data and our ability to account for virtually all PPI use, because the drugs are not sold over-the-counter in Canada. However, some limitations of our study merit emphasis. We had no access to serum magnesium levels, and the validity of hospital coding for hypomagnesemia using administrative databases is unknown. Second, our results derived from patients aged 66 years and older, and the generalizability to younger patients is unknown. Third, we cannot account for intermittent PPI use, although this would be expected to attenuate the association with hypomagnesemia. Finally, clinically significant cases of hypomagnesemia may have been missed in our analysis, particularly those presenting with arrhythmias or other metabolic abnormalities such as hypokalemia.

On the basis of the large number needed to harm (approximately 76,591), our study should not discourage clinicians from prescribing PPIs to appropriate patients. While routine screening of serum magnesium concentrations in patients on PPIs is likely unwarranted, clinicians should be aware of this association, particularly in patients on long-term PPI therapy and those with hypokalemia or associated cardiac or neurologic symptoms. In patients with hypomagnesemia in the setting of PPI therapy, we suggest reassessing the need for ongoing therapy and considering other treatment options.

In summary, we found an association between outpatient PPI therapy and hospitalization with hypomagnesemia among older patients. Our findings highlight what may be an underappreciated adverse effect of PPI therapy. Future research may help further characterize the significance of this effect, and the importance of cumulative dose and duration of PPI therapy. In the interim, we suggest that physicians recognize the potential causative role of PPIs in patients with hypomagnesemia, and reconsider PPI therapy in such patients.

## Supporting Information

Text S1 STROBE statement.
(DOC)

## Abbreviations

IQR: interquartile range
PPI: proton-pump inhibitor
TRPM: transient receptor potential melastin
